# InGaAsP/InP Nanocavity for Single-Photon Source at 1.55-μm Telecommunication Band

**DOI:** 10.1186/s11671-017-1898-y

**Published:** 2017-02-20

**Authors:** Hai-Zhi Song, Mukhtar Hadi, Yanzhen Zheng, Bizhou Shen, Lei Zhang, Zhilei Ren, Ruoyao Gao, Zhiming M. Wang

**Affiliations:** 1 Institute of Fundamental and Frontier Sciences, University of Electronic Science and Technology of China, Section 2-4, Jianshebei Road, Chengdu, 610054 Sichuan China; 2 School of Optoelectronic Information, University of Electronic Science and Technology of China, Section 2-4, Jianshebei Road, Chengdu, 610054 Sichuan China; 3 Department of Fundamental Laser-Optoelectronic Technology, Southwest Institute of Technical Physics, Section 4-7, Renminnan Road, Chengdu, 610041 Sichuan China

**Keywords:** Nanocavity, Single-photon source, Quantum dot, Distributed Bragg reflector, 78.67.-n, 78.67.Hc, 78.67.Pt

## Abstract

A new structure of 1.55-μm pillar cavity is proposed. Consisting of InP-air-aperture and InGaAsP layers, this cavity can be fabricated by using a monolithic process, which was difficult for previous 1.55-μm pillar cavities. Owing to the air apertures and tapered distributed Bragg reflectors, such a pillar cavity with nanometer-scaled diameters can give a quality factor of 10^4^–10^5^ at 1.55 μm. Capable of weakly and strongly coupling a single quantum dot with an optical mode, this nanocavity could be a prospective candidate for quantum-dot single-photon sources at 1.55-μm telecommunication band.

## Background

Optical microcavities and nanocavities are widely studied for their prospects in many fields of research and technology, such as optical communication, nonlinear optics, optoelectronics, and quantum information technology [1, 2]. For solid-state quantum information processing, microcavities and nanocavities containing semiconductor quantum dots (QDs) have been demonstrated to be effective as indispensable devices such as efficient [3–5] and indistinguishable single-photon sources (SPSs) [6, 7] and coherent quantum-control devices [8, 9]. Among many cavity types, pillar cavities are advantageous for fiber-based quantum information processing owing to high coupling efficiency to fiber [10] and suitability for electrical driving [11]. For the purpose of quantum communication over a silica fiber-based network, 1.55-μm InAs/InP QDs are promising as SPSs [12] and thus pillar cavities containing InP-based QDs are strongly required. Like the pillar cavities for InAs/GaAs QDs, the straight way for InAs/InP QDs might be micro- or nanopillar cavities composed of InP lattice-matched distributed Bragg reflector (DBR) layers such as InP/InGaAsP and AlInGaAs/AlInAs, which might be monolithically fabricated. However, this kind of pillar cavity is thought to be so high, due to small refractive index contrast of ~0.2 [13] that nobody wants to try. By increasing the refractive index contrast in DBRs, people tried pillar cavities hybridizing semiconductor and dielectric materials, e.g., Ta_2_O_5_/SiO_2_–InP [14] and Si/SiO_2_–InP [15, 16]. However, the hybrid approach is not ideal due to the complicated fabrication process, defects near the light source caused by thin active layer, and mismatching thermal expansion in different materials. Consequently, a practically good pillar cavity has not been available yet as a SPS applied in 1.55-μm quantum information processing. More efforts must thus be devoted to finding methods of overcoming the above-stated problems. We are herewith considering some techniques beyond material hybrid. In the case of planar DBR cavity, an effective way to increase refractive index contrast of InP-based materials is to introduce air gaps by sacrificing some semiconductor layers [13, 17]. For pillar cavities, partial air-gap layers like in GaAs/air DBR cavities [18] might be incorporated to enhance the refractive index contrast. In this work, therefore, we propose a nanopillar cavity consisting of InGaAsP/InP layers with partial air gaps, which can be monolithically fabricated. It is presented that this nanocavity has high quality (*Q*) factors and small mode volumes, satisfying the requirements of SPS at 1.55-μm telecommunication band.

## Methods

**Fig. 1:**
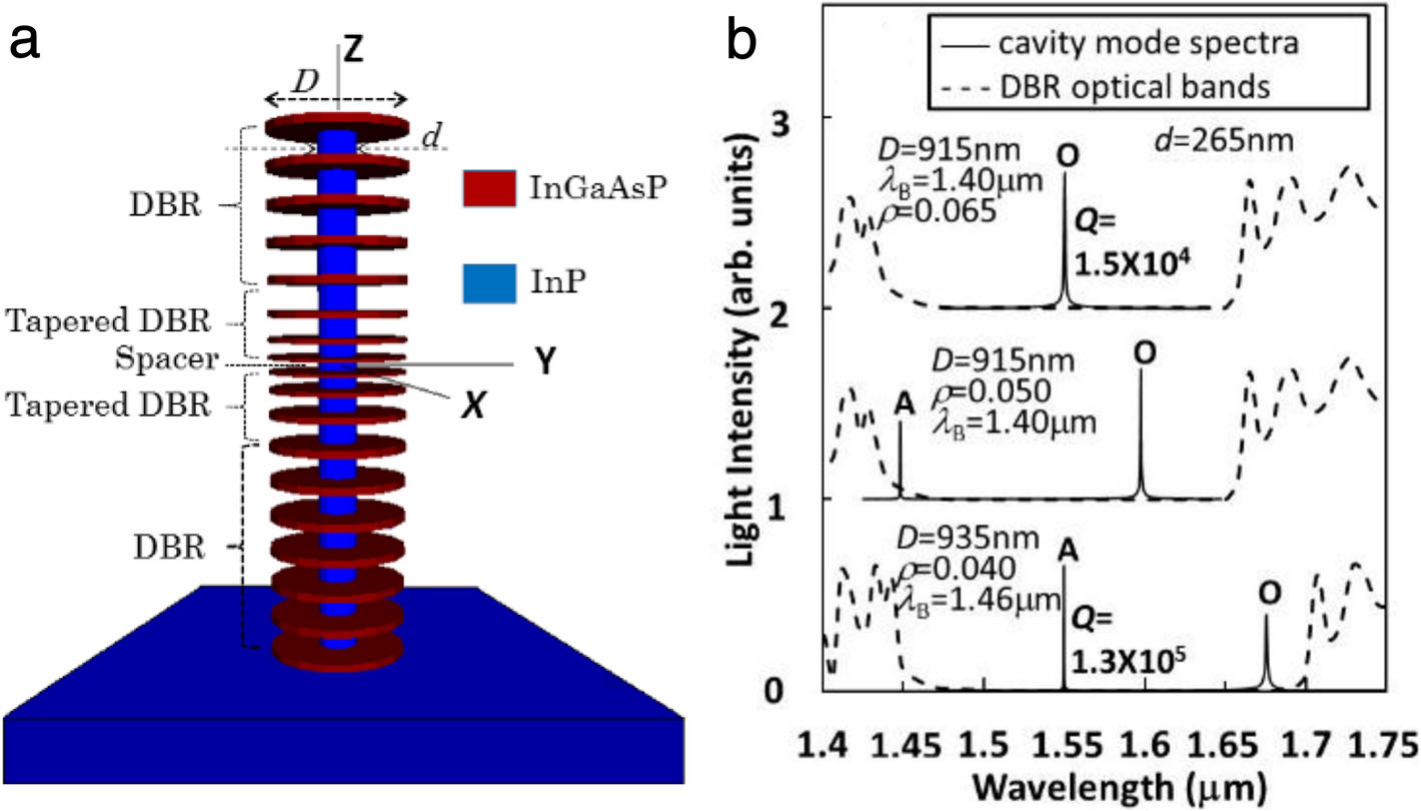
**a** Three-dimensional schematic demonstration of the proposed cavity. **b** Examples of the calculated optical mode spectra with corresponding DBR optical bands. In **b**, the lines for different design conditions are vertically shifted for clarity

The proposed cavity structure is schematically demonstrated in Fig. 1a. It shows that disk shaped (in the XY plane) and coaxially set (in the Z direction) InGaAsP and InP layers with different diameters *D* and *d*, respectively, are alternatively stacked on an InP substrate. Effectively, the small-sized InP layers are compassed by surrounding air gaps, or namely with air apertures. The InGaAsP layers are lattice matching to the InP substrate and have an energy gap larger than the photon energy of 1.3-μm wavelength, so that they are extremely transparent for ~1.55-μm light. Compared to the previous air-gap DBR cavities [13, 17, 18], in which non-air-gap regions are imperfect features or mechanical supporters, the remaining semiconductor in the partial air-gap layers here takes both the mechanically supporting and optically confining roles so that the present cavity appears completely free standing.

In more detail, the top and bottom parts of the cavity are conventional DBRs composed of periodical InGaAsP and InP layers. Each InP layer in the DBRs is set as thick as *t*_1_ = *λ*_B_/4, where *λ*_B_ is the Bragg wavelength, set to be around 1.55 μm. This thickness is actually a quarter wavelength of air because the optical media of this layer in the pillar is mainly air rather than InP. In the case of planar air-gap DBR cavities [13, 17, 18], semiconductor layers are usually set to be three-quarter-wavelength thick, but our simulation implies that this design in our case hardly has good cavity quality. Thus, the InGaAsP layers in the DBRs are set quarter-wavelength thick, i.e., *t*_2_ = *λ*_B_/(4*n*_2_), where *n*_2_ is the refractive index of InGaAsP. Inserted between the conventional DBRs are more InGaAsP/InP-air-aperture segments (pairs) as tapered DBRs on both the top and bottom sides. Here, “taper” means adiabatically deducing the layer thicknesses as the DBR extends towards the cavity center (spacer) [19, 20]. In detail, the tapered DBRs have linearly decreasing layer thicknesses *t*_1*i*_ = *t*_1_(1−*ρ*(2*i*−1)) for InP and *t*_2*i*_ = *t*_2_(1−2*ρi*) for InGaAsP, where *i* stands for the taper segment number and *ρ* is the tapering slope of layer thickness, i.e., the decreased fraction per tapered layer. In between the tapered DBRs, an InP layer is inserted as the spacer layer with thickness *t*_0_ = *t*_1_(1−2*ρN*), where *N* is the total taper segment number in one tapered DBR. An InAs QD is set in this layer as the light source.

We calculated the optical properties of this cavity, e.g., the cavity mode spectra, the DBR optical band structure, the optical field distribution of a mode, and the *Q* factor, using finite-difference time-domain method with the optical constants of the materials cited or deduced from Ref. [21]. All these simulations were performed using a commercial tool Rsoft.

## Results

It is found that the proposed cavity is of high quality when it has 4/6.5 pairs of InGaAsP and InP layers in the top/bottom DBRs and *N* = 3 segments in the tapered DBRs. The pillar of this cavity structure appears some 7–8-μm high, which is the same as the high-quality Si/SiO_2_–InP hybrid pillar cavity [16] and the high-quality GaAs/AlGaAs monolithic pillar cavity [19]. By massively trying out different cavity geometrical parameters *D*, *d*, *λ*_B_, and *ρ*, the optical properties were systematically studied. When *d* = 265 nm, *D* = 915 nm, *λ*_B_ = 1.40 μm, and *ρ* = 0.065, it is observed that the optical mode with the longest wavelength, i.e., the fundamental cavity mode peaks at 1.550 μm with *Q* factor of 1.5 × 10^4^, as shown by the topmost spectrum in Fig. 1b. This is surprising because a similarly high InGaAsP/InP DBR pillar cavity exhibits *Q* factor of only a hundred or so. It indicates that the air apertures resolve the problem of low refractive index contrast in InP-based pillar cavities. We label this fundamental mode as mode O hereafter. It is roughly at the center of the DBR stopband, corresponding to an optimized condition. As shown by the middle spectrum in Fig. 1b, when *ρ* is tuned to be 0.05, mode O shifts to a longer wavelength with *Q* factor decreasing to 6000. Meanwhile, there arises a new mode near the shorter stopband edge. Further changing *ρ* to be lower, the new mode, termed mode A hereafter, shifts towards the middle of stopband and its *Q* factor increases, while mode O is shifting towards the longer stopband edge with smaller and smaller *Q* factor. The lower spectrum in Fig. 1b shows that, by changing all the three parameters *D* = 935 nm, *ρ* = 0.04, and *λ*_B_ = 1.46 μm, we obtain an optimized mode A, peaking at 1.550 μm and with *Q* factor of 1.3 × 10^5^. This *Q* factor is one order of magnitude higher than the optimized mode O and even higher than that of a typical Si/SiO_2_–InP hybrid pillar cavity with a similar pillar size [16]. At the same time, mode O becomes a peak near the longer stopband edge with *Q* factor below 3000.

**Fig. 2:**
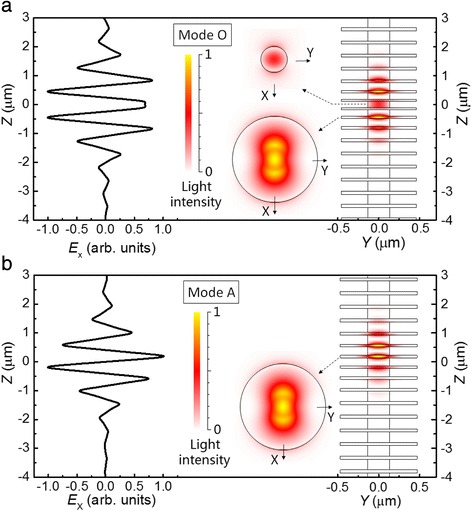
Line mode profiles along vertical Z direction and patterned mode profiles on the YZ and XY planes of the optimized optical **a** mode O and **b** mode A. Note that the line profiles are indicated by the X-polarized electric field, while the plane profiles are displayed by light intensity. The *thin lines* on the light intensity patterns show the outlines of the optimized cavities

It is worth stressing that the proposed cavity is a nanometer-scaled pillar structure since the cavity lateral size, especially the air-aperture diameter, is less than 1 μm. The direct result of this nanoscale is the small mode volume *V*, which is 1.08 and 0.94(*λ*/*n*)^3^ for optimized modes O and A, respectively, where *λ* is the mode wavelength and *n* is the refractive index at the point of maximal light intensity. The light intensity distribution, which determines the mode volume through integrating over the cavity [1], is shown in Fig. 2. We note that both modes are twofold degenerate with orthogonal main polarizations, but for simplicity, we arbitrarily select X as the main polarization direction to describe their properties. The colored patterns tell us that the light fields of both modes O and A are laterally well confined within the semiconductor cavity media, i.e., quite weak in the air, and vertically confined mainly within the tapered DBRs and the spacer region. This resulted from the air apertures, which tend to compact the light fields laterally into an area with diameter *d* and vertically into a few DBR layers by increasing the reflective rate of DBRs.

The vertical distribution patterns in Fig. 2 also show the effect of adiabatic design in the tapered DBRs. That the light intensity extends over a few segments implies gentle confinement of light fields, which provides a reasonable explanation for the high *Q* factors [22] in both Modes O and A. What is more important, there really exists a large difference in the two modes. Mode O has strong light fields both in the small-diameter InP spacer and the large-diameter InGaAsP layers, while mode A leaves its optical field mainly in the InGaAsP layers. With sub-wavelength lateral size *d* < *λ*_B_/*n*, the InP spacer with air aperture is more subjective of leaking through the side wall. This is likely the reason why the optimized mode A has higher *Q* than mode O. In terms of the Z-dependent line profile of the main electric field along the X direction, *E*_X_, mode O is symmetric, whereas mode A is antisymmetric to the cavity central plane *Z* = 0. This might be understood by a mode coupling between two fundamental modes corresponding to two differently sized nanopillars, as could bring in some new modes like bonding (symmetric) and antibonding (antisymmetric) states, since a present cavity looks like a mixture of two pillars with different sizes.

To use a cavity with optimized mode A, there seems a problem that the light source in the InP spacer has a weak interaction with the mode field due to minimum field intensity in the spacer. As a matter of fact, 1.55-μm InAs QD can also be settled in the InGaAsP layer [23]. In addition, we find that exchanging InGaAsP and InP layers can give similarly good cavity properties.

**Fig. 3:**
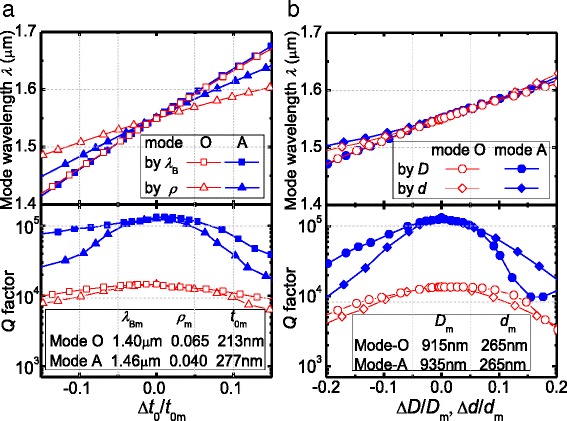
Varying mode wavelength *λ* and *Q* factor as the **a** vertical and **b** lateral sizes deviate from the optimized conditions for both modes O and A. Note that the vertical sizes, i.e., the layer thicknesses, are tuned by *λ*_B_ or *ρ* but displayed in terms of the spacer thickness *t*_0_. The lateral sizes are tuned by cavity diameter *D* or air-aperture diameter *d*

Although we have obtained high *Q* factors on cavities with some special designs, it is necessary to further investigate the dependence on cavity design parameters, because the practical fabrication process may not be that exact. First, let us look at the dependence on vertical size. For simplicity, we here characterize the effects of the tuning parameters *λ*_B_ and *ρ* in terms of the variation in spacer thickness Δ*t*_0_ = *t*_0_-*t*_0m_, where *t*_0m_ is the optimized spacer thickness, since both of them give rise to changes in the spacer thickness *t*_0_. As shown in Fig. 3a, the mode wavelength *λ* varies linearly with the relative change in layer thickness at different rates. The tapering slope *ρ* has less effect on *λ* because it causes a more local geometric change in the cavity. Conservatively speaking, *λ* remains within 1.55 ± 0.05-μm range as the layer thickness changes within ±5%. To this degree of thickness deviation, the *Q* factors of modes O and A do not degrade a lot but remain over 1.3 × 10^4^ and 10^5^, respectively, although they would decay almost by a factor of 3 and 10 with thickness deviation of ±15%. In more detail, *λ*_B_ degrades *Q* factor more weakly than *ρ* does because an entire geometric change by *λ*_B_ remains, to a high degree, the mode profile, while a local geometric change by *ρ* breaks the mode profile. By the way, mode O is more robust than mode A, since its *Q* factor remains over 10^4^ with thickness change of ±10%. Given that *t*_0_ is typically over 100 nm, ±5–10% precision means a layer-thickness control error within ±5–10 nm, which is rather easy in modern epitaxial techniques enabling controllability at atomic layer level.

Now, let us examine the dependence on lateral size. Figure 3b shows the variations in the cavity properties versus the relative change in cavity diameter Δ*D/D*_m_ = (*D*−*D*_m_)/*D*_m_ and in air-aperture diameter Δ*d/d*_m_ = (*d*−*d*_m_)/*d*_m_, where *D*_m_ and *d*_m_ are the optimized *D* and *d* values, respectively. It is normal that smaller lateral scale results in a shorter mode wavelength *λ* due to more localized geometrical confinement. A slight difference lies in the dependences of mode wavelength *λ* on *D* and *d*. The weakly sub-linear change with *D* resembles conventional pillar cavities and can be explained by waveguide dispersion [24]. The super-linear dependence on *d* might be related to the existence of air apertures. Anyway, *D* and *d* have influences on *λ* to the similar extent in both modes O and A. It stays within 1.55 ± 0.05 μm as the lateral sizes deviate by up to ±10%. As to the *Q* factor, its degradation with *D* and *d* deviations, caused by deviated effective incident angle of light on the DBRs [10], is as much as that with thickness deviations. In a more quantitative view, ±5% change in *D* or *d* keeps mode O almost of no degradation and mode A over 10^5^ in *Q* factor. Again, mode O seems a little more robust than mode A, since ±8% deviation in lateral dimensions can keep its *Q* factor over 10^4^. For a typical diameter of ~200 nm, the lateral size precision of ±5–10% means an error within ±10–20 nm. This degree of controllability has been already available in the state-of-the-art nanotechnology [25]. The robustness against the uncertainty of the fabrication process implies the high technical feasibility to fabricate high-quality nanopillar cavity at 1.55-μm telecommunication band.

Remember that in some cases, mode O and mode A coexist. Within the above tuning ranges, however, the main mode always stands with much higher *Q* factor than the other. There thus will be no serious interference from the useless mode when the main mode is working. By the way, *Q* versus *D* in mode A appears a little abnormal, i.e., *Q* rising back as *D* becomes large enough. It may result from coupling with higher order optical modes as mentioned in the previous pillar cavities [15, 26].

## Discussion

With the simulated high quality, the proposed InGaAsP/InP-air-aperture nanopillar cavity is hopefully a candidate for 1.55-μm QD-SPSs. Let us analyze now how the application aspects of the proposed nanocavity would be.

Above all, the likeliness of single-photon emission is enhanced by the nanoscale of the cavity. A single InAs/InP QD is a good single-photon emitter under usual excitation conditions [12] since the excitation pulse duration can easily be in the order of picoseconds or less, much shorter than the exciton lifetime of nanosecond order. Isolating a single QD is thus sufficient for single-photon emission. Supposing a high QD density of 6 × 10^10^ cm^−2^ and a good inhomogeneous width of ~50 meV, it is easy to get that there will be less than 1 QD resonant to a 1.55-μm cavity mode with *Q* factor of 10^4^ (i.e., mode width less than 0.08 meV) in a pillar cavity with a diameter of 1 μm. This property highly guarantees the single-photon nature of an InAs/InP QD SPS composed of this nanopillar cavity. In addition, the nanoscaled structure is also beneficial to incorporating SPSs into a photonic integration chip, which is necessary in the future quantum information processing network.

With *Q*/*V* over 3000, the Purcell factor of a weak coupling cavity [27], simply speaking the enhancement degree of spontaneous emission of the light source, is estimated to be more than 100. This degree can reduce the spontaneous emission lifetime of InAs/InP QDs (a few ns [28]) to be shorter than the coherence time (~100 ps [29]). It suggests that a present cavity with optimized mode O (*Q* ~ 10^4^ and *V* ~ 1(*λ*/*n*)^3^) could be used as a photon-indistinguishable SPS [6] and GHz operating SPS at 1.55-μm band. In the case of strong coupling, which enables coherent transfer of quantum states between a light emitter and a cavity mode, theoretical criteria [30] suggest that *Q*/*V* ^1/2^ > 10^4^ is more than enough for a 1.55-μm InAs/InP QD emitter [16]. A present cavity with optimized mode A (*Q* ~ 10^5^, *V* < 1(*λ*/*n*)^3^) is thus able to realize coherently controllable single-photon devices at 1.55-μm band.

As compared to other pillar cavities, the nanocavity proposed here takes some advantages. The widely used GaAs/AlGaAs DBR pillar cavity can also be of high quality *Q* > 10^5^ and nanometer size [19]. If it is applied for 1.55-μm band, however, the pillar would be ~16-μm high, much more difficult to fabricate than the present ~7-μm high pillar. Furthermore, it is hard to contain 1.55-μm QDs in GaAs-based DBR structures. Therefore, a GaAs/AlGaAs pillar cavity is less usable as a 1.55-μm SPS than the present one. As to the InP-based materials, our calculations suggest that a conventional InGaAsP/InP DBR pillar cavity with a nanometer-scaled diameter can work with *Q* factor of ~10^4^ and Purcell factor of ~100 at 1.55 μm, able to weakly couple a single InAs/InP QD with a cavity mode. However, there should be ~40/70 periods of DBRs, meaning a pillar too high (~30 μm) to be currently producible. It is thus clear that, even only for weak coupling, InP-based DBR pillar cavity is much less suitable than the present one. The hybrid pillar cavities, such as Ta_2_O_5_/SiO_2_–InP [14] or Si/SiO_2_–InP [15, 16], are subject to interface defects and different thermal expansion coefficients. The present cavity consists of only InP-based epitaxial materials so that it is free of interface defects and thermal expansion difference. More significantly, it can be fabricated by a monolithic process, e.g., epitaxial growth for the multilayer structure, dry etching to form a pillar and selective wet-etching to develop air apertures. The fabrication process is obviously simpler and of lower cost than the techniques for fabricating hybrid pillar cavities [14]. Although just slightly different from the processes of previous air-gap DBR cavities [13, 17, 18], which always introduce finite size effect and the influence from the peripheral supporters, this process can make cavity size highly exact so that the simulation-predicted cavity quality could be more achievable than the previous air-gap cavities.

There may arise a problem that the good cavity quality we have obtained may have a distance from that of a real cavity [18], because the above fabrication process could be not well determined to make an ideal cavity structure. One aspect may be that, with size less than 1 μm, process-induced surface roughness might degrade the cavity quality by, e.g., edge scattering [31]. At present, however, InP-based nanocavities, e.g., 100~400 nm-sized photonic crystal cavities, have readily exhibited practical *Q* factors above 10^4^ [32] although surface roughness does exist. On the other hand, the present advanced techniques allow controlling sidewall surface roughness of InP-based nanostructures to be less than 1 nm while remaining of good optical quality [33, 34]. Primitive calculations on our nanocavities suggest that a sidewall roughness of 1 nm degrades the *Q* factor by 5–10%. There may be another aspect that the chemical etching influences the optical quality by introducing surface states [35]. Nevertheless, recent researches demonstrate that, when some suitable echant and/or surface passivation are used, a wet-etched InP-based nanopillar presents nice optical properties [36]. A sophisticated wet-etching process would have only a minor effect on the quality of a nanocavity [37]. Furthermore, it is worth noting that InP/InGaAsP materials have a relatively low surface recombination velocity than GaAs/AlGaAs materials [38], suggesting much better practical quality of our cavity than that of the GaAs/air DBR ones [18]. Consequently, a real cavity as here proposed could be expected to have optical quality very close to what is designed here.

We see that the InGaAsP/InP-air-aperture nanopillar cavity proposed here is of high quality for weak and strong coupling, able to give single photons from InAs/InP QDs, producible by a monolithic process, robust against process uncertainty, and thus better than conventional GaAs/AlGaAs, InP/InGaAsP, and hybrid pillar cavities. It is therefore prospective as a candidate for 1.55-μm QD SPSs applied in silica fiber-based quantum information system.

## Conclusion

In this work, we proposed a new structure of InP-based nanopillar cavity as QD SPS at 1.55-μm telecommunication band. A design of air apertures resolves the problem of small refractive index contrast in InP-based DBR pillars, so that the cavity can be fabricated using a monolithic process, which was difficult for previous 1.55-μm pillar cavities. The air apertures and the tapered DBRs bring about pillar diameter less than 1 μm, small mode volume of ~ (*λ*/*n*)^3^, and high *Q* factor of 10^4^–10^5^. The properties of this cavity well satisfy the requirements of efficient/photon-indistinguishable and coherently controllable 1.55-μm QD SPSs. Further with the quality robustness against process uncertainty, this InGaAsP/InP-air-aperture nanopillar cavity is prospective as the SPS for 1.55-μm silica fiber-based quantum information processing.
